# Detection of Pathogenic Mycobacteria Based on Functionalized Quantum Dots Coupled with Immunomagnetic Separation

**DOI:** 10.1371/journal.pone.0020026

**Published:** 2011-05-27

**Authors:** Emmanouil Liandris, Maria Gazouli, Margarita Andreadou, Leonardo A. Sechi, Valentina Rosu, John Ikonomopoulos

**Affiliations:** 1 Department of Animal Science and Aquaculture, Agricultural University of Athens, Athens, Greece; 2 Laboratory of Biology, School of Medicine, University of Athens, Athens, Greece; 3 Department of Biomedical Science, Microbiology, University of Sassari, Sassari, Italy; University of Delhi, India

## Abstract

Mycobacteria have always proven difficult to identify due to their low growth rate and fastidious nature. Therefore molecular biology and more recently nanotechnology, have been exploited from early on for the detection of these pathogens. Here we present the first stage of development of an assay incorporating cadmium selenide quantum dots (QDs) for the detection of mycobacterial surface antigens. The principle of the assay is the separation of bacterial cells using magnetic beads coupled with genus-specific polyclonal antibodies and monoclonal antibodies for heparin-binding hemagglutinin. These complexes are then tagged with anti-mouse biotinylated antibody and finally streptavidin-conjugated QDs which leads to the detection of a fluorescent signal. For the evaluation of performance, the method under study was applied on *Mycobacterium bovis* BCG and *Mycobacterium tuberculosis* (positive controls), as well as *E. coli* and *Salmonella* spp. that constituted the negative controls. The direct observation of the latter category of samples did not reveal fluorescence as opposed to the mycobacteria mentioned above. The minimum detection limit of the assay was defined to 10^4^ bacteria/ml, which could be further decreased by a 1 log when fluorescence was measured with a spectrofluorometer. The method described here can be easily adjusted for any other protein target of either the pathogen or the host, and once fully developed it will be directly applicable on clinical samples.

## Introduction

Most members of the genus *Mycobacterium* are harmless microbes that live in diverse soil and aqueous environments; however, there are a number of pathogenic species that affect humans and animals causing mainly tuberculosis, leprosy, and paratuberculosis [1]–[4].

Despite their medical and environmental importance, mycobacteria have always proven difficult to identify. This is due to a combination of factors, principal among them being their low growth rate and fastidious nature. Therefore the application of molecular biology methods was exploited from very early for the detection of mycobacteria. However, incorporation of DNA amplification techniques in routine diagnosis requires highly-specialised personnel, dedicated equipment and space. The latter is applied within the context of the vigorous precautions needed to avoid the “carry over effect” (successive passage of amplicons from one test sample to the other) that especially for the polymerase chain reaction (PCR) can easily lead to false positive results even in the presence of minute amounts of target DNA.

An alternative approach that might resolve the problems mentioned above relies on the incorporation of nanotechnology to the development of novel diagnostic tests. In recent years, many nanosystems have been utilized for pathogen detection [5]–[7]. Semiconductor quantum dots (QDs) or nanocrystals have emerged as a very promising class of fluorophores [8], [9]. Unlike conventional organic dyes, QDs can be excited by a wide spectrum of wavelengths, they have great photostability, and their emission spectra, which differ according to size and material composition, are narrow, symmetrical, and tunable. With these characteristics, QDs have minimal interference from natural autofluorescent particles and can be used in the multiplex detection of different molecular targets in various biological specimens [9].

Recently we developed two prototypical diagnostic assays designed for use at point-of-care. These methods incorporate gold nanoparticles [10] and a combination of magnetic beads (MBs) and cadmium selenide QDs [11] for the detection of conserved genomic regions of DNA belonging to *Mycobacterium* spp. without the need of amplification. Here we present the first stage of the development of the latter of these methods for the detection of mycobacterial surface antigens using streptavidin-conjugated QD together with biotinylated anti-*Mycobacterium* spp. polyclonal antibody.

## Materials and Methods

### Antibodies

The following antibodies were incorporated in the assay under study: Two murine monoclonal antibodies against the *Mycobacterium tuberculosis* heparin-binding hemagglutinin (HBHA) (4A8 and 1G10, Icosagen Srl, Estonia). A biotinylated polyvalent antibody produced in rabbit against *Mycobacterium tuberculosis* PPD, which according to the manufacturer reacts with related mycobacterial species (BP2027B, Acris Germany). A sheep anti-mouse biotinylated antibody (R1256B, Acris, Germany).

### Conjugation of MBs with anti-Mycobacterium antibodies

Stretavidin coated MBs (dynabeads M-280, Invitrogen, USA) were functionalized with the biotinylated polyvalent antibody mentioned above. For this purpose, 40 µg of antibody were added to 200 µl (10 mg/ml) streptavidin coated MBs and incubated at room temperature for 30 min. For the removal of unbound antibody, conjugated MBs were washed 5 times with PBS with the aid of a magnetic device (Dynal MPC-s, Invitrogen, CA, USA) and dissolved in 200 µl of PBS containing 0.1% BSA.

### Functionalization of QDs with streptavidin

Cadmium selenide (CdSe) QDs (15–20 nm in size) with a maximum emission wavelength of 655 nm, shelled with ZnS and a polymer coating presenting carboxylic groups were purchased by Invitrogen (Q21321MP, Invitrogen, USA). QDs were coated with streptavidin prior to use according to the manufacturer's instructions. Briefly, 50 µl of QDs were diluted in 400 µl of borate buffer 10 mM (pH 7.4), 96 µl of streptavidin solution (10 mg/ml) (Invitrogen, CA, USA) and 11.4 µl of EDC (10 mg/ml) (Sigma-Aldrich, MO, USA) and were incubated at room temperature for 90 min. Streptavidin coated QDs were washed 5 times with 500 µl of borate buffer 50 mM (pH 8.3) on an Amicon Ultra-4 Filter (Millipore, MA, USA) and dissolved into 50 mM borate buffer (pH 8.3) to a final volume of 500 µl.

### Bacterial strains

For the evaluation of performance, the method under study was applied on *Mycobacterium bovis* BCG (Pasteur) and *Mycobacterium tuberculosis* (n = 3 isolates) (positive controls) that were grown on Middlebrook 7H11 (BD, USA) with OADC (Oleic acid, Albumin, Dextrose, Catalase) enrichment (BD, USA) and 0.4% pyruvate (Sigma-Aldrich, Germany). Negative controls consisted of *E. coli* (n = 2 isolates) and *Salmonella* spp. (n = 4 isolates) that were grown on Brain Heart Infusion Agar (Oxoid, UK) for 24 h.

### Limit of Detection

For the assessment of the limit of detection (LOD) of each method we used duplicate solutions of *Mycobacterium bovis* BCG. Two series of ten-fold dilutions ranging from 10^8^ to 10 cells/ml were prepared using standard bacteriological techniques. Briefly, a few fresh colonies grown on solid media were suspended in sterile screw cup glass tubes containing PBS and 6 glass beads. The suspensions were mixed with a vortex for a few seconds in order to break large clumps and were left to settle for 5 mins. The number of bacteria in the suspension was determined with the aid of a Petroff Hauser counting chamber. The LOD was defined as the lowest concentration level that could be determined to be statistically different from the negative controls (Analytical detection limit guidance 1996).

### Bacterial detection

**Figure 1:**
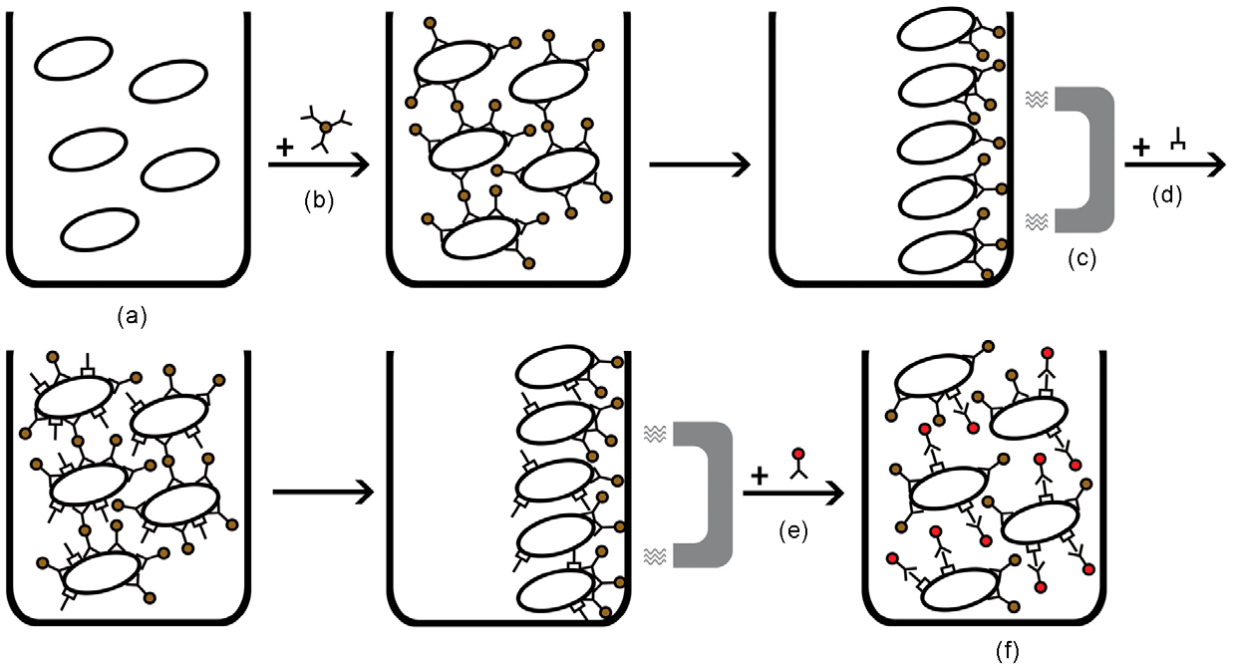
Bacterial cells (a) are separated from the matrix using MBs coupled with genus specific polyclonal antibodies (b) and a magnetic device (c). These complexes are then tagged with anti-HBHA (d) and anti-mouse biotynylated antibody and streptavidin-conjugated QDs (e) which lead to the detection of a fluorescent signal (f).

The principle of the assay is based upon the separation of bacterial cells using MBs coupled with genus-specific polyclonal as well as the mouse monoclonal antibodies mentioned above. These complexes are then tagged with anti-mouse biotinylated antibody and finally streptavidin-conjugated QDs which lead to the detection of a fluorescent signal (Figure 1). To this purpose, 1 ml of each dilution of the bacteria that comprised positive and negative controls or an equal volume of PBS BSA 0.1% (blank) were coupled at first with 40 µl of genus specific polyclonal antibody conjugated MBs, then with 1 µg (500 ng each) of HBHA monoclonal antibody, then 10 µl (0.2 mg/ml) of an anti-mouse biotinylated antibody (R1256B, Acris, Germany), and finally 40 µl of streptavidin coated QDs. For each hybridization step the dilutions were incubated at 37°C for 20 min with gentle agitation, separated by the matrix with the aid of a magnetic device (Dynal MPC-s, Invitrogen, CA, USA), washed once with PBS BSA 0.1% and then re-suspended in 200 µl PBS 0.1% BSA. For the direct visual observation of fluorescence, samples were transferred on a UV transilluminator with UV emission at 312 nm (Vilber Loumat, France). Fluorescence was also detected with a spectrofluorometer (excitation 480 nm emission 655 nm, PMT 450V), (Infinite M200, Tecan USA) and with an epifluorescence microscope with a 50 W mercury lamp (AXIOSKOP 40, Carl Zeiss Light Microscopy Germany) using the following filter set 460SPUV/475DCXRU/D655/40 nm (Chroma Technology, USA).

## Results

### Conjugation of MBs with anti-Mycobacterium antibodies

**Figure 2:**
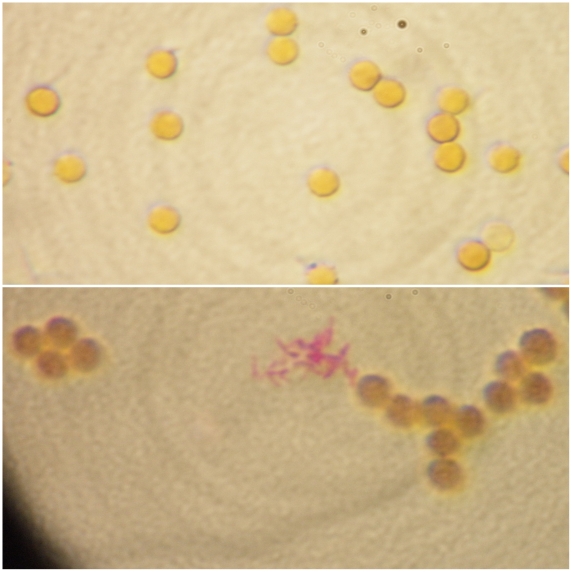
Comparison of the ability of free (A) and antibody conjugated (B) magnetic beads to capture BCG bacilli. The detection of acid fast bacteria was possible only in the second case. (Zhiel Neelsen stain 1000X).

Assessment of the immobilization of antibodies on the surface of MBs was performed by spectrophotometric measurement at 280 nm of the antibody solution before and after conjugation with nanoparticles (data not shown). Confirmation on the same issue was provided by the microscopic observation of Ziehl-Neelsen stained preparations of MBs with and without the antibodies that were used for the immunocapture of BCG cells. As shown in Figure 2 the observation of bacterial cells closely associated with MBs was possible only in the case of those coupled with antibodies while in the absence of antibody no bacteria were observed.

### Bacterial detection/Limit of Detection

**Figure 3:**
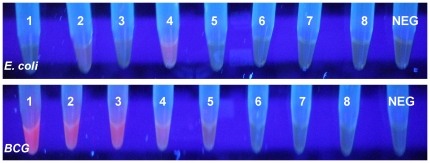
Results recorded by the proposed method for *E. coli* and *M. bovis* BCG serial dilutions ranging from 10^8^to 10^1^ cfu/ml (numbers 1–8) and negative controls. Fluorescence is evident in BCG samples with concentration up to 10^4^ cfu/ml.

The direct visual observation of blanks and negative controls did not reveal any fluorescent signal (Figure 3) as opposed to the mycobacteria pathogens reported above that reacted positively at or above the LOD concentration.

**Figure 4:**
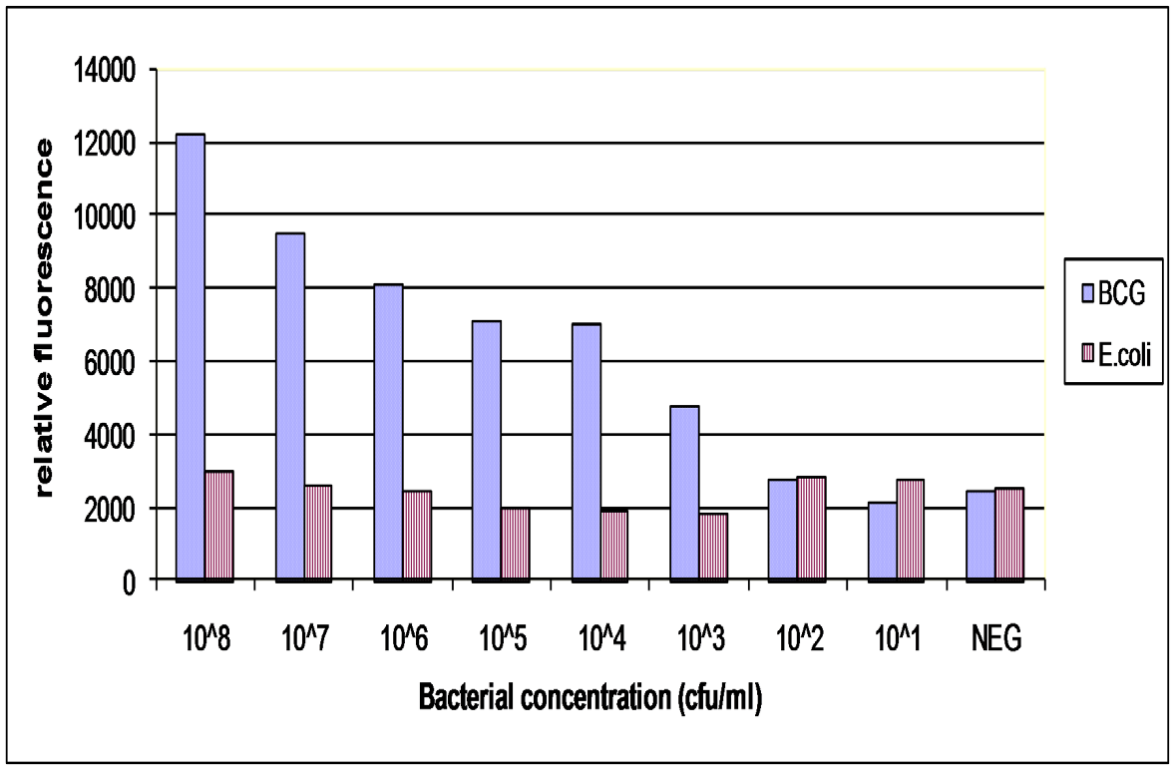
Relative fluorescence values of BCG and *E. coli* serial dilutions.

The LOD of the methodology described here was defined to 10^4^ bacteria/ml of sample when results were assessed by visual observation of the test tubes (Figure 3). The detection limit of the method could be further decreased to 10^3^ bacterial cells/ml when the fluorescence of the samples was measured with a spectrofluorometer (Figure 4).

**Figure 5:**
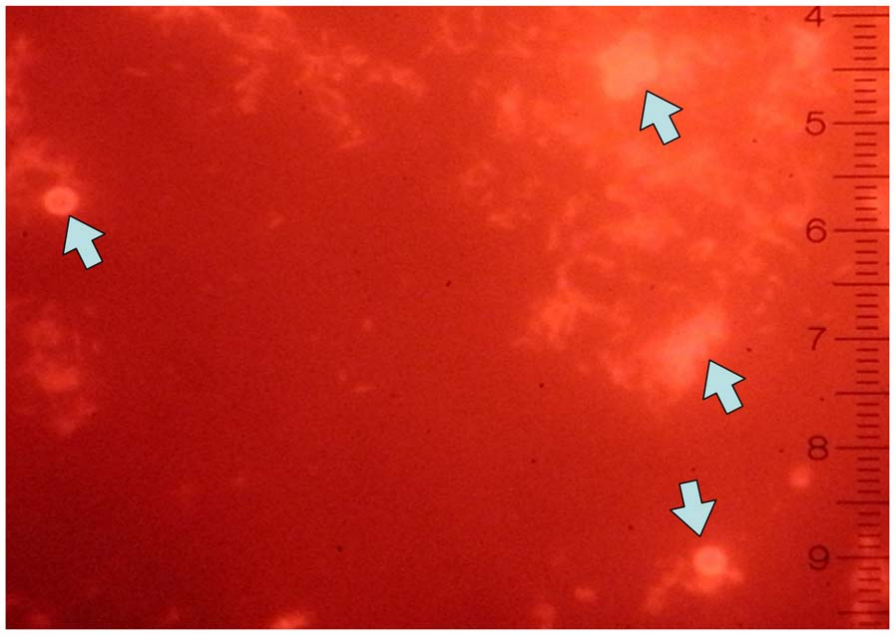
MBs/bacteria/QDs complexes visualized by fluorescent microscopy. Magnetic beads (arrows) are surrounded by a fluorescent halo with shape and dimensions compatible with mycobacteria. 1000X.

In order to perform a validation of the read-out, microscopic slide smears were prepared from the samples (negative controls and serial dilutions of the positive controls) and they were examined by fluorescence microscopy. This revealed the presence of magnetic beads surrounded by a fluorescent halo with dimensions comparable to those of mycobacteria only for the positive and for none of the negative controls (Figure 5).

## Discussion

Previously we presented a method incorporating CdSe QDs for the detection of mycobacterial DNA that is not prone to false results associated with PCR-inhibitors or the carry over effect since it does not involve amplification of DNA [11]. The same method that proved its efficiency on clinical samples is adapted here for the detection of mycobacterial surface antigens. In this case the methodology is simplified in that it does not require sample processing for DNA isolation, which facilitates its incorporation at point-of-care. The use of QDs in the proposed approach bypasses the disadvantages of fluorescent dyes often incorporated into immuno-detection tests such as rapid photobleaching, narrow excitation spectrum and low signal intensity.

The evidence provided here indicate that streptavidin-conjugated CdSe QDs can specifically bind to the mycobacterial pathogens incorporated in this study after their separation using antibody coupled MBs. The procedure is very easy to perform and does not require any dedicated equipment. Combined with a simple spectrophotometer the suggested approach allows direct detection of 1000 bacterial cells/ml of sample, which can be further improved by the use of alternative or multiple protein targets and brighter QDs.

The method under study was designed around mycobacterial HBHA antigen. This is a 28-kDa, methylated surface-associated protein that mediates the interaction of the tubercle bacilli with the host. HBHA acts as an adhesin for non-phagocytic cells such as epithelial, playing an important role in the systemic extra-pulmonary dissemination of *Mycobacterium tuberculosis* [12], [13]. Expectedly HBHA has been under investigation towards the development of vaccines and tests as a significant immuno-reactive protein and a clinical indicator of tuberculosis [14], [15].

Owing to the evident significance of HBHA it was considered a good candidate for the development of the method under study that was designed to target the protein itself rather than the immune response to it. The latter which would have simplified the procedure significantly, was not selected for this stage of development, since our aim was to demonstrate that the specific approach can support detection of mycobacteria in a reliable manner using at the same time clinically significant indicators. For reasons of applicability the method described here incorporates a combination of mycobacterial polyclonal and monoclonal antibody that increases specificity and at the same time facilitates its adjustment for any other protein target, either of the pathogen or the host. Evidently the assay can be easily extended to the detection of any other bacterium as the adaptation of the method would require only the incorporation of specific antibodies for the pathogen in question.

Once fully developed, the method described here will be directly applicable on clinical samples in the context of the one previously reported [11]. Combined with the latter, the proposed application will allow detection of both DNA and protein targets in a simple manner using the same methodology with different types of nanoprobes, without dedicated instrumentation apart from a magnetic device and a UV lamp.
